# Implementation of a Routine Screening Program for Latent Tuberculosis Infection among Patients with Acute Leukemia at a Canadian Cancer Center

**DOI:** 10.3390/curroncol29120731

**Published:** 2022-11-29

**Authors:** Rbab Taha, Sagar Kothari, Farid Foroutan, Melissa Gitman, Vikas Gupta, Tram Nguyen, Coleman Rotstein

**Affiliations:** 1 Immunocompromised Host Infectious Diseases Service, Ajmera Transplant Center, University Health Network, Toronto, ON M5G 2N2, Canada; 2 Department of Medicine, Faculty of Medicine, University of Toronto, Toronto, ON M5S 3H2, Canada; 3 Ted Rogers Centre for Heart Research, University Health Network, Toronto, ON M5G 2N2, Canada; 4 Department of Health Research Methods, Evidence and Impact, McMaster University, Hamilton, ON L8S 4L8, Canada; 5 Department of Pathology, Molecular and Cell-Based Medicine, Icahn School of Medicine at Mount Sinai, Mount Sinai Hospital, New York, NY 10029, USA; 6 Division of Medical Oncology, Princess Margaret Cancer Centre, University Health Network, Toronto, ON M5G 2C1, Canada

**Keywords:** latent tuberculosis infection, tuberculin skin testing, leukemia

## Abstract

Background: Screening for latent tuberculosis infection (LTBI) in patients with hematological malignancy is recommended because of their increased risk of tuberculosis (TB). We assessed the utility of tuberculin skin test (TST) screening in patients with acute leukemia and subsequent outcomes of LTBI treatment. Methods: We retrospectively evaluated patients ≥16 years of age with acute leukemia from 2013–2014 with a TST planted and read prior to the initiation of antineoplastic chemotherapy treatment. Demographics, clinical information and treatment outcomes of LTBI therapy were compared between patients with positive TST (≥10 mm induration) and negative TST. Results: A total of 389 patients with acute leukemia were included in the cohort. Of them, 37/389 (9.5%) had a positive TST. Only 3.4% (8/235) of individuals originating from North and South America as well as the Caribbean were TST positive, while 21% (20/95) of individuals from Asia were TST positive. Diagnostic imaging findings consistent with prior tuberculosis infection were higher in TST positive patients compared to TST negative ones (29.7% versus 9.4%, *p* < 0.0001). Furthermore, 31/38 patients (81.6%) who were TST positive received LTBI therapy, which was well tolerated. There was no significant difference in overall survival among those who received LTBI therapy compared to those who did not. No patients developed active TB. Conclusions: Universal screening with TST may be of low yield in individuals with acute leukemia unless patients originate from a TB endemic country. When therapy for LTBI is prescribed, patients with acute leukemia do not experience drug-induced liver toxicity and are likely to complete the intended duration of therapy, thus preventing the development of active tuberculosis.

## 1. Introduction

As reported by the World Health Organization, approximately 9.9 million people (95% uncertainty interval: 8.9 to 11 million) developed illness with tuberculosis (TB) in 2020 [1]. This was equivalent to 127 (uncertainty interval: 114–140) cases per 100,000 inhabitants worldwide and represented a slight decline compared to 2019, probably due to disruptions in TB services with the COVID-19 pandemic. Eight countries (India, China, Indonesia, the Philippines, Pakistan, Nigeria, Bangladesh, and South Africa) accounted for two thirds of the global disease burden. In Canada, the incidence rate of active TB is considerably lower with rates of 4.8 and 4.9 per 100,000 population in 2016 and 2017 respectively and continues to be stable with a rate of 4.7 per 100,000 in 2020 [2,3]. However, the majority of TB cases (73.5%) in Canada in 2020 originated in foreign-born individuals [4]. In the province of Ontario, the incidence of TB between the years of 2012 to 2015 ranged from 4.4 to 4.6 per 100,000 population, comparable to the overall incidence in Canada during that time frame [4,5].

In patients with cancer, the risk of developing TB was found to be 9 times higher compared to the risk in individuals in the general population in the United States [6]. In a recent meta-analysis involving 23 studies, the highest risk of TB in cancer patients was noted amongst patients with hematologic malignancies with an incidence risk ratio of 26 [7]. Indeed, acute leukemia confers a high cumulative incidence rate per 100,000 among patients with hematologic malignancies, and TB reactivation may cause considerable morbidity with delays in chemotherapy as well as mortality [7,8]. As a result, a strategy of screening for latent tuberculosis infection (LTBI) has been recommended in patients with hematological malignancies. Accordingly, the Public Health Agency of Canada has recommended screening for LTBI in all patients with hematological malignancies and those who undergo stem cell transplantation [9]. Screening patients for LTBI provides an opportunity for early detection and subsequent treatment, curtailing the morbidity and mortality associated with TB in patients with hematologic malignancies.

There are, however, potential impediments to the success of screening. First, the sensitivity of the tuberculin skin test (TST) may be low in patients with hematologic malignancies due to their immunosuppression, which confounds the diagnosis of LTBI [10]. In contrast, false positive test results may expose patients to unnecessary therapy, and lead to delays in the initiation of necessary antineoplastic chemotherapy [11]. Furthermore, some reports have concluded that prophylaxis with isoniazid (INH), the most common agent used for LTBI treatment, does not decrease the risk of TB in patients with hematological malignancies [10]. There is a paucity of data to support TST sensitivity and validity as a reference standard for LTBI diagnosis in patients with hematological malignancies [10].

INH for 9 months has remained the mainstay of LTBI therapy with an estimated efficacy of 90% in immunocompetent individuals who complete therapy [12]. Concerns over polypharmacy and additive side effects have prompted reluctance to complete LTBI therapy in patients with hematologic malignancies [8,13].

The purpose of this study was to evaluate the TST positivity rate in patients with acute leukemia (acute myeloid and lymphoblastic leukemia (AML and ALL)) and assess the outcomes of treatment for LTBI and the utility of performing this screening strategy universally for patients with AML and ALL. We also describe the concordance of TST screening with computerized tomography (CT) imaging of the chest consistent with a diagnosis of granulomatous disease. We further describe the frequency and outcomes of LTBI treatment in patients with acute leukemia who have undergone antineoplastic chemotherapy.

## 2. Methods

### 2.1. Study Design

We performed a retrospective cohort test-negative study on patients ≥16 years of age with newly diagnosed acute leukemia at the Princess Margaret Cancer Centre in Toronto, Ontario, Canada, between 1 January 2013 and 3 December 2014. This healthcare facility only cares for adult patients ≥16 years old. Leukemia diagnosis was corroborated based on bone marrow aspirate morphology, flow cytometry, and molecular markers. Consecutive patients with electronic medical records were included if they had a TST planted prior to or at the start of chemotherapy, using 5 tuberculin units of purified protein derivative (Tubersol, Sanofi Pasteur Limited, Toronto, ON, Canada) and read within 48–72 h [14]. Patients were excluded if they had known active TB, declined treatment, or did not receive chemotherapy for their leukemia and received palliative supportive care only. The standard procedure at our institution was to perform and interpret the results of a TST prior to starting antineoplastic chemotherapy for patients with newly diagnosed acute leukemia in accordance with standardized techniques [14].

Clinical information collected for each patient included: demographic information, country of origin, underlying diagnosis, baseline chest imaging findings upon admission to the facility based on a radiologist’s interpretation, dates of TST performance with site of implantation and size of induration if at all when read, LTBI treatment regimen, all antineoplastic chemotherapy administered and purpose of this chemotherapy (whether for induction, consolidation, or reinduction for relapse or refractory disease), and duration of LTBI therapy. Bacille Calmette–Guerin (BCG) vaccination status as well as date of its administration were recorded if available. The influence of BCG on positive TST was only considered if such immunization with BCG had occurred <10 years prior to TST positivity [15,16].

The successful outcome of LTBI treatment was determined by the absence of active tuberculosis after receiving LTBI therapy. The safety of LTBI treatment was assessed based on the discontinuation rate of therapy due to side effects, and elevations in liver enzymes above baseline values. It should also be noted that data collection for safety was truncated at the time of the last follow up which may have occurred prematurely prior to the scheduled completion of therapy. Details regarding the cause of mortality whether attributable to leukemia or alternative etiologies were not recorded. This study protocol was approved by the Research Ethics Board of the University Health Network, Toronto, ON, Canada.

### 2.2. LTBI Case Definition

A case of LTBI was defined as those individuals who had a 5-tuberculin-unit TST planted subcutaneously and developed ≥10 mm of induration at the time of reading 48–72 h later [9]. Diagnostic imaging (CT scan or X-ray of the chest) findings had to be consistent with prior granulomatous disease, including apical fibrosis, lung nodules, calcified granulomas, and pleural scarring. TB was diagnosed by the demonstration of acid-fast bacilli on modified Ziehl–Nielsen staining or positive molecular PCR testing for *M. tuberculosis* complex DNA in clinical samples using Gene Xpert MTB/RIF (Cepheid Inc., Sunnyvale, CA, USA) as reported by the Public Health Laboratory of Ontario, Toronto, ON, Canada.

### 2.3. Statistical Analysis

TST positive individuals were compared to test negative controls by means of descriptive statistics. For continuous variables, if normally distributed, the *t*-test was performed and for skewed continuous variables, the Kruskal Wallis test was used. The Chi-square test was employed to compare categorical variables, unless the count of one group was less than 5, in which case Fisher’s exact test was used. In addition, those individuals who received therapy for LTBI were evaluated for completion of therapy by virtue of 6–9 months of therapy. Outcomes were described for all treated patients including reasons why treatments were stopped. The Kaplan Meier estimator was undertaken to report on the overall survival of groups within the cohort (GraphPad Prism version 9.4.1, GraphPad Software, San Diego, CA, USA). Patient group survival was compared by the log rank test. Statistical significance was set at *p* < 0.05.

## 3. Results

### 3.1. Selection of Cohort

**Figure 1:**
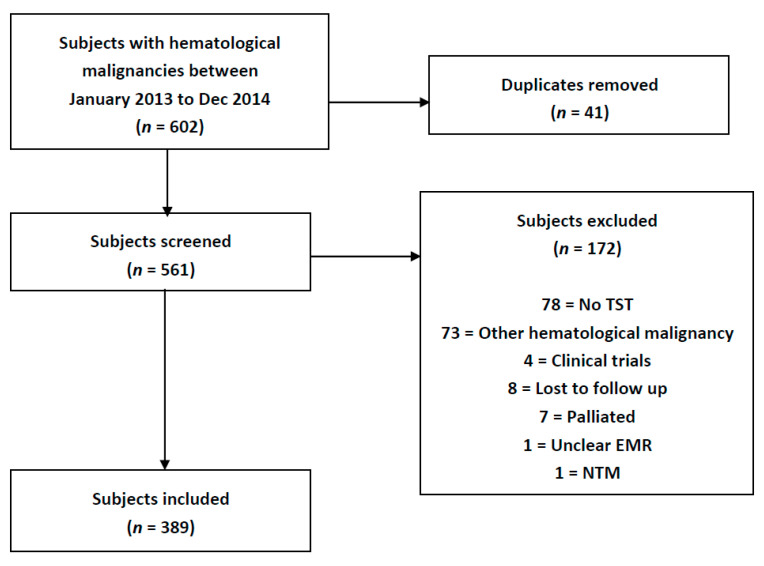
Study Flow Diagram.

From January 2013 to December 2014, 602 patients with acute leukemia were identified through the Leukemia Service clinic intake lists at Princess Margaret Cancer Centre in Toronto, ON, Canada. After duplicates were removed, 561 patients were screened for inclusion. The following individuals were excluded: 78 patients did not have a TST placed or read, 73 patients had a hematological malignancy other than acute leukemia, 8 patients were lost to follow up, 4 patients were on clinical trials with drugs of an unknown mechanism of action, 7 patients were designated for palliation, 1 patient had an unclear medical record and 1 patient had a non-tuberculous mycobacterium identified (see Figure 1). A total of 389 patients were included, of which 308 had a diagnosis of AML and 81 ALL.

### 3.2. Cohort Characteristics

The demographics of those patients included in the study and categorized by TST positive and negative results are demonstrated in Table 1. No significant differences were noted in age and sex between those participants who were TST positive vs. TST negative. TST positivity was noted in 3.4% (8/235) of those individuals from North and South America and the Caribbean as compared to 21.1% (20/95) of those from Asia (*p* < 0.001). Thus, individuals from Asia were most likely to be TST positive (54.1%), while those from North and South America and the Caribbean were more likely to be TST negative (64.5%) (*p* < 0.001). The most common chemotherapeutic regimens employed were cytarabine and daunorubicin for induction (67.6% in the TST positive vs. 51.1% in the TST negative groups), high-dose cytarabine for consolidation (43.2% vs. 41.5%), and fludarabine-containing regimens for re-induction (16.2% vs. 17.0%). Azacitidine was used as induction chemotherapy in elderly individuals and those with myelodysplastic syndrome and excess blasts.

### 3.3. Chest Imaging and TST Concordance

A CT scan of the chest was performed on 93% (364/390) of the patients. Of the 17 individuals who had chest X-ray imaging, all were TST negative and none had imaging findings compatible with LTBI (5 with consolidation, 1 with a pulmonary opacity and 11 with no findings). TST positive patients were more likely to have chest CT scan findings of apical fibrosis, calcified granulomas, and scarring compared to those individuals with a negative TST (29.7% vs. 9.4% respectively; *p* < 0.0001). However, the finding of consolidation in the chest imaging (17.6% vs. 2.7%) was more prevalent in TST negative patients.

### 3.4. LTBI Therapy

A total of 38 were TST positive, of whom 31 (81.6%) were given LTBI therapy. Of the 6 patients who did not receive therapy, 1 patient had received BCG vaccination, and for 5 patients, the reason for lack of administration of therapy was not determined.

We noted that 6 patients were TST negative yet, received LTBI therapy. All 6 of these patients had CT scan findings compatible with LTBI, such as apical fibrosis or calcifications, with or without consolidation, with 4 patients being from Asian countries and 2 from the Caribbean. In addition, 5 of these 6 patients had TST induration of 6–9 mm, while the last had an induration of 0 mm. Moreover, one of these patients had received vaccination with BCG.

The most common LTBI treatment regimens was isoniazid (INH) and vitamin B6 (VitB6) in both TST negative (*n* = 6, 100%) and positive (*n* = 27, 87.1%) patients, whereas, 2 patients received INH-VitB6-rifampin and 1 patient received rifampin alone. One patient initially received INH-VitB6 and later had therapy changed to moxifloxacin due to hepatotoxicity. The median duration of treatment was 198 days (IQR: 105–275 days), and 21 (56.8%) completed therapy. The only adverse event noted was hepatotoxicity (elevation in liver transaminases above baseline). A total of 8 patients (21.6%) experienced hepatoxicity and 3 of these patients had therapy halted; 5 patients had therapy stopped due to palliation or death, while 8 patients had therapy stopped for an unknown reason.

### 3.5. Outcomes

**Figure 2:**
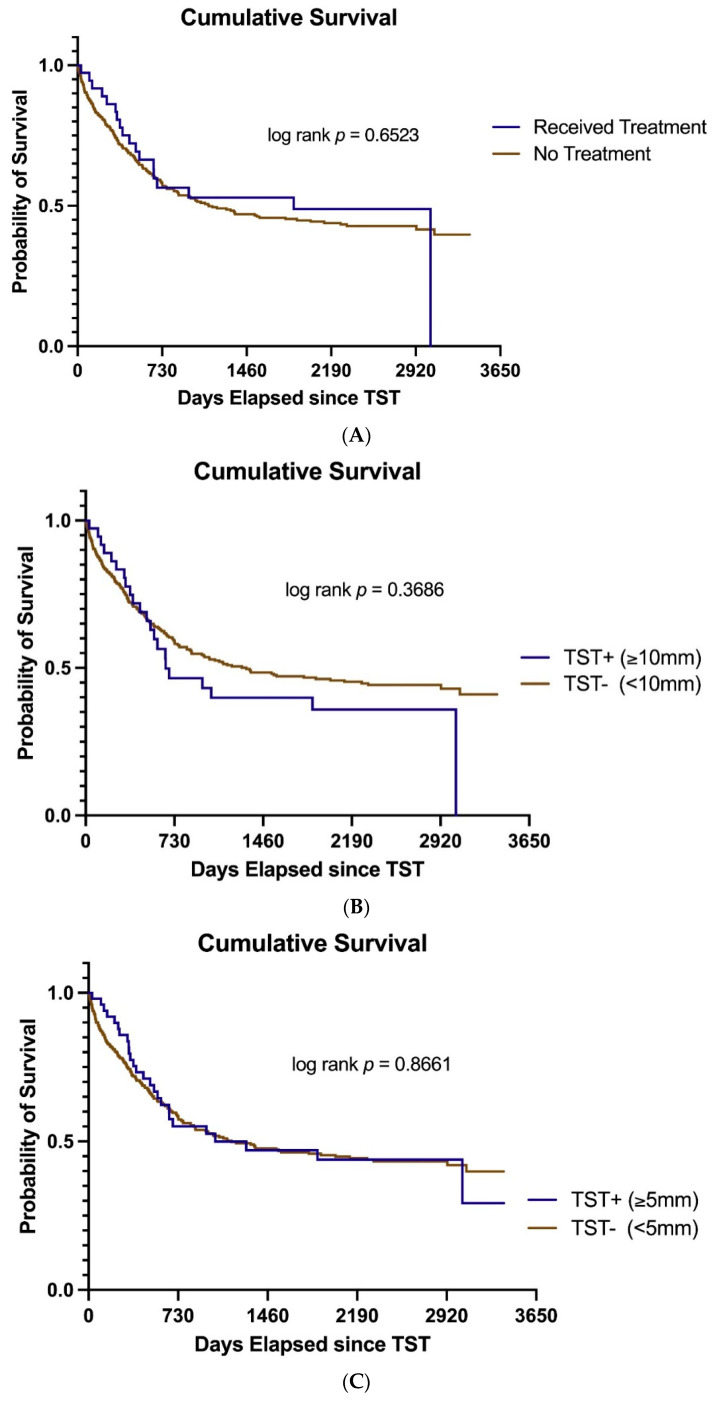
Cumulative survival. (**A**) Overall survival in 37 patients with acute leukemia who received LTBI therapy compared to 352 patients who were not treated. (**B**) Overall survival in the 37 patients who were TST positive (≥10 mm of induration, *n* = 37) compared to those who were TST negative (*n* = 352). (**C**) Overall survival in patients with TST of ≥5 mm of induration (*n* = 51) compared to those with negative TST (<5 mm of induration, *n* = 338).

The overall survival among those individuals who received LTBI therapy (37 patients) compared to those who did not (352 patients) is illustrated in Figure 2A and was not significantly different (log rank *p* = 0.652). In addition, we also assessed the survival of those study participants who were TST positive with ≥10 mm of induration (*n* = 37) compared to those classified as TST negative (<10 mm of induration, *n* = 352) in Figure 2B and once again there was no significant difference (log rank *p* = 0.3686). Finally, we then performed a sensitivity survival analysis for all individuals with a TST showing ≥5 mm of induration (*n* = 51) compared to those with a TST < 5 mm of induration (*n* = 338) and again this showed no significant difference in survival (Figure 2C, log rank *p* = 0.866). One must be cognizant that patients with AML most often died of their primary malignancy. No patients with either TST positive or negative status developed active TB.

## 4. Discussion

Tuberculosis produces morbidity and mortality that has a global impact. This is especially pertinent for cancer patients with leukemia. This retrospective study comprehensively analyzed the outcomes of and utility of TST testing in patients with acute leukemia. We were struck by the relative low frequency of positive TST (37/389, 9.5%), although individuals originating from Asia predominated in this group (54.1% see Table 1). Thus, the yield of performing TST was higher in patients who originated from TB endemic countries as mentioned above. Second, we found that conventional LTBI prophylaxis (INH for 6–9 months) was well tolerated by leukemia patients and most patients were able to complete treatment (56.8% completion rate).

The prevalence of positive TST in our cohort was 9.5%. In comparison, Richeldi and colleagues noted a 10.5% prevalence among 95 patients with hematologic malignancy. However, our cohort was considerably larger and was more homogenous as it involved only patients with acute leukemia. In contrast, Orsorio-Lopez and colleagues demonstrated a higher prevalence of 31.2% for LTBI infections when focusing primarily on non-Hodgkin’s and Hodgkin’s lymphoma patients who comprised 347 of 446 (77.8%) of the patients with hematologic malignancies [17]. They considered a positive TST to be 5 mm of induration and observed that 250 out of 446 (56.1%) patients were vaccinated for BCG [17]. Moreover, only 34 patients were diagnosed with leukemia. Our study robustly focused on individuals with acute leukemia while considering 10 mm of induration to constitute a positive TST with only 3 individuals with BCG vaccination [9]. Moreover, even when we adjusted our analysis to include those individuals with ≥5 mm of induration, once again there was no difference in survival in our cohort whether one was TST positive or negative.

Only 8 individuals (21.6%) in our cohort experienced elevation in liver transaminase tests above baseline values but therapy was only discontinued in 3 patients. All of these, liver transaminase test abnormalities were less than five times the upper limit of normal. Only one of these patients was switched to an alternative agent. Once again, these data echo those of Sanchez-Garcia and colleagues who studied toxicity of LTBI treatment in patients with hematologic malignancies [18].

Utilizing CT scans of the chest for the diagnosis of LTBI did not augment our ability to diagnose LTBI compared to TST. We did observe some congruency of CT scan findings compatible with previous TB and positive TST results, but such findings were only noted in 29.7% of patients with a positive TST. In contrast, when the TST was negative only 9.4% of patients were categorized as possessing chest CT scan findings compatible with previous tuberculosis. Thus, there was a 3-fold increase in concordance of TST and CT findings in the group with a positive TST compared with those individuals with a negative TST. However, one must be cognizant that CT scan findings compatible with previous TB do not possess high specificity as other clinical entities may mimic these findings.

Our review of TST and therapy for LTBI was noteworthy in that none of our patients developed active TB during the follow up period of up to 8 years. Although the patients’ underlying leukemia may have led to their early demise abrogating the need for follow up, 54.1% of the patients treated for LTBI still had 2 years of follow up (Figure 2).

One may also raise concern that BCG vaccination may have falsely increased our positive TST numbers. We did not believe that this concern was warranted, as the time interval from vaccination to leukemia diagnosis with TST performance was greater than 10 years. As demonstrated by others, BCG vaccination has little influence on TST positivity with a 10 year or more time lapse after vaccination [15,16].

Our study has several limitations. First, this study is a single-center retrospective observational cohort evaluating data that are 8 years old. Our center has a diverse patient population with patients originating from countries of high TB endemicity. Our TST prevalence findings may not be directly generalizable to centers with a different patient makeup. Second, it is unclear what role acute leukemia with the concomitant pancytopenia had in predisposing patients to falsely negative TST. Interferon gamma release assay (IGRA) testing has better sensitivity in detecting previous tuberculosis exposure in patients with hematological malignancies as reported by Richeldi and colleagues (17.9% to 26.3% vs. 10.5% for TST) but may also be hampered by the pancytopenia present in acute leukemia [10]. Unfortunately, IGRA testing was not undertaken in our healthcare facility due to its higher costs. Furthermore, 14% of the 561 patients screened did not have TST performed and were therefore excluded from our analysis.

Overall, our data show that TST universal screening may be of low yield in individuals with acute leukemia unless patients are members of a high-risk population, such as those with acute leukemia who were born in a TB endemic country. Thus, it may be prudent to focus TB screening on such individuals rather than employing universal screening. In addition, once therapy for LTBI is initiated, patients with acute leukemia should not experience drug-induced liver toxicity. Indeed, they are likely to complete the intended duration of therapy, thus preventing the development of active tuberculosis.

## Figures and Tables

**Table 1:** Cohort demographics stratified by positive PPD (≥10 mm).

	TST− (*n* = 352)	TST+ (*n* = 38)	*p*-Value
**Age, Mean (SD)**	55.1 (16.6)	56.4 (13.1)	0.583
**Sex, Female (%)**	163 (46.3%)	15 (40.5%)	0.62
**Continent**			
Africa	6 (1.7%)	4 (10.8%)	<0.001
Asia	75 (21.3%)	20 (54.1%)	
Europe	28 (8.0%)	4 (10.8%)	
North and South America, Caribbean	227 (64.5%)	8 (21.6%)	
Missing	16 (4.5%)	1 (2.7%)	
**Diagnosis**			
ALL	76 (21.6%)	5 (13.5%)	0.294
AML	276 (78.4%)	32 (86.5%)	
**Induction Chemotherapy**			
ATRA-containing regimen	17 (4.8%)	0 (0%)	0.258
Azacitidine	11 (3.1%)	1 (2.7%)	
Cytarabine	4 (1.1%)	1 (2.7%)	
Cytarabine + Anthracycline	1 (0.3%)	0 (0%)	
Cytarabine + Daunorubicin	180 (51.1%)	25 (67.6%)	
Fludarabine-containing regimen	39 (11.1%)	4 (10.8%)	
None	21 (6.0%)	0 (0%)	
Tyrosine-kinase inhibitors	2 (0.6%)	1 (2.7%)	
Vincristine-containing regimen	75 (21.3%)	5 (13.5%)	
Vincristine-containing regimen+ Tyrosine-kinase inhibitors	2 (0.6%)	0 (0%)	
**Consolidation Chemotherapy**			
ATRA-containing regimen	16 (4.5%)	0 (0%)	0.543
Azacitidine	6 (1.7%)	1 (2.7%)	
Azacitidine + Allopurinol	1 (0.3%)	0 (0%)	
Blinatumomab	1 (0.3%)	0 (0%)	
Cytarabine (high-dose)	146 (41.5%)	16 (43.2%)	
Fludarabine-containing regimen	35 (9.9%)	8 (21.6%)	
Hydroxyurea	1 (0.3%)	0 (0%)	
None	71 (20.2%)	6 (16.2%)	
Tyrosine-kinase inhibitors	2 (0.6%)	0 (0%)	
Vincristine-containing regimen	71 (20.2%)	6 (16.2%)	
Vincristine-containing regimen+ Tyrosine-kinase inhibitors	2 (0.6%)	0 (0%)	
**Re-Induction Chemotherapy**			
ATRA-containing regimen	3 (0.9%)	0 (0%)	0.613
Azacitidine	12 (3.4%)	4 (10.8%)	
Blinatumomab	8 (2.3%)	0 (0%)	
Cytarabine	8 (2.3%)	1 (2.7%)	
Cytarabine + ATRA-containing regimen	1 (0.3%)	0 (0%)	
Cytarabine + Daunorubicin	5 (1.4%)	0 (0%)	
Fludarabine-containing regimen	60 (17.0%)	6 (16.2%)	
None	246 (69.9%)	25 (67.6%)	
Tyrosine-kinase inhibitors	3 (0.9%)	0 (0%)	
Vincristine-containing regimen	6 (1.7%)	1 (2.7%)	
**Chest Imaging Conducted**			
Computerized Tomography	327 (92.9%)	37 (97.4%)	0.496
X-ray	17 (4.8%)	0 (0%)	
None	8 (2.3%)	0 (0%)	
**Chest Imaging Findings**			
Apical Fibrosis	10 (2.8%)	3 (8.1%)	0.0015
Apical Fibrosis, Calcified Granuloma	1 (0.3%)	1 (2.7%)	
Apical Fibrosis, Consolidation	6 (1.7%)	1 (2.7%)	
Apical Fibrosis, Lung Nodules	1 (0.3%)	1 (2.7%)	
Calcified Granuloma	9 (2.6%)	5 (13.5%)	
Calcified Granuloma, Consolidation	3 (0.9%)	0 (0%)	
Calcified Granuloma, Pleural Scarring	1 (0.3%)	0 (0%)	
Calcified Lymph Node, Consolidation	1 (0.3%)	0 (0%)	
Calcified Lymph Node, Ground-Glass Opacity	1 (0.3%)	0 (0%)	
Consolidation	60 (17.0%)	1 (2.7%)	
Consolidation, Lung Nodules	2 (0.6%)	0 (0%)	
Ground-Glass Opacity	34 (9.7%)	7 (18.9%)	
Ground-Glass Opacity, Lung Nodules	1 (0.3%)	0 (0%)	
Hydropneumothorax	1 (0.3%)	0 (0%)	
Lung Nodules	26 (7.4%)	1 (2.7%)	
None	187 (53.1%)	15 (40.5%)	
Opacities	8 (2.3%)	2 (5.4%)	
Apical Fibrosis	10 (2.8%)	3 (8.1%)	0.0015
**Treatment for Latent Tuberculosis Infection**			
No Treatment	346 (98.3%)	6 (16.2%)	<0.001
Treatment	6 (1.7%)	31 (81.6%)	
**Relapses of Underlying Disease**			
None	243 (69.0%)	26 (70.3%)	1
1 Relapse	106 (30.1%)	11 (29.7%)	
2 Relapses	2 (0.6%)	0 (0%)	
3 Relapses	1 (0.3%)	0 (0%)	
**Development of Active TB**	0 (0%)	0 (0%)	<0.001
**Treatment for Latent Tuberculosis Infection**			
Isoniazid + Vitamin B6	6 (100%)	27 (87.1%)	1
Other *	0 (0%)	4 (12.9%)	

* Other included: isoniazid + vitamin B6 + rifampin (*n* = 2), rifampin (*n* = 1) and isoniazid + vitamin B6, then moxifloxacin (*n* = 1).

## Data Availability

The data presented in this study are available upon reasonable request from the corresponding author.

## Abbreviations

AML, acute myelogenous leukemia; ALL, acute lymphoblastic/lymphocytic leukemia; BCG, Bacille Calmette–Guerin; CT, computerized tomography; INH, isoniazid; LTBI, latent tuberculosis infection; TB, tuberculosis; TST, tuberculin skin test.
